# The Metal Neurotoxins: An Important Role in Current Human Neural Epidemics?

**DOI:** 10.3390/ijerph14121511

**Published:** 2017-12-05

**Authors:** Keith Schofield

**Affiliations:** Materials Research Laboratory, University of California Santa Barbara, Santa Barbara, CA 93106-5121, USA; KSHome@ucsb.edu; Tel.: +1-805-966-6589

**Keywords:** neurotoxicants, biomarker body levels, minimum risk levels (MRLs), pregnancy, over-burdens, synergism, vaccines, fish diets, risk factors

## Abstract

Many published studies have illustrated that several of the present day neurological epidemics (autism, attention deficit disorder, Alzheimer’s) cannot be correlated to any single neurotoxicant. However, the present scientific examination of the numerous global blood monitoring databases for adults that include the concentrations of the neurotoxic elements, aluminum (Al), arsenic (As), lead (Pb), manganese (Mn), mercury (Hg), and selenium (Se) clearly indicate that, when considered in combination, for some, the human body may become easily over-burdened. This can be explained by changes in modern lifestyles. Similar data, solely for pregnant women, have been examined confirming this. All these elements are seen to be present in the human body and at not insignificant magnitudes. Currently suggested minimum risk levels (MRL) for humans are discussed and listed together with averages of the reported distributions, together with their spread and maximum values. One observation is that many distributions for pregnant women are not too dissimilar from those of general populations. Women obviously have their individual baseline of neurotoxin values before pregnancy and any efforts to modify this to any significant degree is not yet clearly apparent. For any element, distribution shapes are reasonably similar showing broad distributions with extended tails with numerous outlier values. There are a certain fraction of people that lie well above the MRL values and may be at risk, especially if genetically susceptible. Additionally, synergistic effects between neurotoxins and with other trace metals are now also being reported. It appears prudent for women of child-bearing age to establish their baseline values well before pregnancy. Those at risk then can be better identified. Adequate instrumental testing now is commercially available for this. In addition, directives are necessary for vaccination programs to use only non-neurotoxic adjuvants, especially for young children and all women of child-bearing ages. Additionally, clearer directives concerning fish consumption must now be reappraised.

## 1. Introduction

The risks associated with neurotoxicant materials have been with us from the age of chemical discovery and particularly through their usage in the colonial and industrial revolution periods. Gold and silver mining casualties, “mad as a hatter” cases, and the Japanese Minamata Bay pollution, all a result of mercury, are historically documented examples [1,2], as well as the possible role for lead in the fall of the Roman Empire. However, these were generally in specific geographic regions or related to industrial consequences. Now, global neurological illnesses that are widespread and cover general populations are becoming increasingly evident in epidemic numbers especially in young children and in the aged, and appear to result from anthropogenic environmental causes undoubtedly coupled to genetic susceptibilities [3,4]. Autism in children affects male/female babies in a ratio of roughly four to one, possibly indicating an additional role of hormones. Overall, its rate of occurrence has increased in the recent period of 30 years by several orders of magnitude. In US births, within the first two years of life, its present rate is at levels of one in eighty births or even more so, and high rates are also of global concern [5,6]. Attention Deficit Hyperactivity Disorder (ADHD) in children has also rapidly grown in recent decades [7]. Alzheimer’s disease has also become globally widespread and is listed as a growing epidemic [8]. Most recently, copper and iron have been suggested as being possibly involved, but this remains speculative due to the fact that these are not known as neurotoxins [9,10]. However, as will be noted, such metals may enhance the effects of some neurotoxicants. In a recent paper involving the Indian population, suggestions center on potential risks from mercury from coal combustion coupled to mercury in marine food products, which is quite plausible [11]. A “dementia epidemic” has also been suggested as being largely under-recognized and in need of concern [12]. Although psychiatric diagnostic abilities certainly have improved and widened the boundaries for most neurological illnesses, the ever drastic changes seen do reflect real growth, yet suggested causes remain unproven and generally remain speculative and not widely accepted. 

One major difference from the past is that significant monitoring capability now exists particularly for chemicals in the body, and there is an increased acceptance and sensitivity to potential environmental contributions and hazards. Moreover, there is a recognition that neurotoxicant materials are present in the human environment and increasing in quantity in some cases through modern lifestyles. Technically, it should, therefore, now be easier in theory to recognize, minimize, and potentially control such risks. In the early 1960s the medical profession became shocked by the effect on some pregnancies of the organic pharmaceutical-chemical thalidomide [13]. Mainly prescribed in Europe, where this was first synthesized, deformed babies were born missing limbs or with other defects. Its causal identity was soon realized and its use banned. However, it emphasized the realization that a fetus in pregnancy had to be considered differently from adults and that toxic substances could be far more life changing to fetal brains and bodies in development. The question of damaging the brain is very different with children, whose brains are still small and forming, whereas adults have fully-developed brains that may be damaged, but in different ways. Additionally, the great diversity seen among people, and in all living species introduces a potential wide range of variations relating to individual genetic susceptibilities that is known to be reflected into toxicity levels [14]. This is undoubtedly true with neurotoxic illnesses, and is quite likely the basis for the breadth of the so-called “spectrum” of neurological illnesses that are observed. This present analysis of medical data, but with a chemical emphasis, is aimed at highlighting the continuing difficulties encountered in the field of toxicology and examines these questions with the aim of better describing present day neurological risks. In this process, it has been noted that although efforts are being made to better manage the human environment, certain social lifestyle changes now are clearly introducing high risk toxicity levels in some cases of several known neurotoxic elements and could easily be the base cause for the observed neurological damage. 

## 2. The Major Neurotoxicants and Their Toxicity

For neurological epidemics, especially if global in nature, the presence of known neurotoxic sources that are commonplace are necessary. Moreover, our knowledge of the body’s strong blood-brain barrier illustrates how effective it is in separating and processing the toxins and neurotoxin chemicals that are normally ingested [15,16]. For many people, this and the body’s detoxification systems are adequate and are automatically sustained throughout life, from conception to death. However, autopsy data are intriguing in establishing that during life, numerous chemicals do accumulate in the brain. In cases of Alzheimer’s disease deaths, a myriad of elements are seen present in addition to the six neurotoxic elements considered herein [17,18]. Due to this, any individual roles are impossible to isolate [19,20,21,22,23]. Whether some of these observed metals are solely sequestered in the brain in benign neutralizing proteins or are in some type of active balance between the body and brain remains unknown. Additionally, such observations are also reported for fetal brain autopsies reflecting similar metals and confirming the mother/baby close inter-connection. Studies have clearly identified the presence of mercury, for example, that correlates directly to the maternal hair level [24]. 

The problem of attaching liability to any epidemic in medicine is generally difficult because of the complex inter-connected chemistry in the human body. In any medical study, the consequences of never fully knowing all the controlling parameters necessarily retains uncertainty for any suggested conclusion. This has been illustrated quite recently, notably in the numerous decade-long US studies with the Seychelles’ and Faroe Island’s peoples, concerning their possible neural effects from having significant fish diets, one based more on whale meat, while the other on ocean fish [25,26,27,28,29]. It now appears that an overlooked role played by selenium as a major brain chelator and the varying natural atomic ratio of selenium to mercury in differing fish varieties may be pivotal for any analysis concerning methylmercury’s true toxicity from a seafood diet [30,31,32,33]. If valid, this will now make any nutritional risk/benefit analysis for dietary fish quite involved and in need of re-assessment. However, as mentioned later, it might resolve observed differences in the data reported between these two large cohort studies for fish-mercury. 

Concerning neurotoxicants, a recent useful study has extensively examined the current medical databases for chemicals that can be listed as developmental neurotoxicants [34]. The produced list contains about 100 molecules or elements drawn from mainly rodent, but also several human, tests. About 85 of the species listed are organic with six organo-metallic chemicals. This then leaves a surprisingly short list of neurotoxic inorganic elements and their compounds that might be considered, especially as these are encountered to varying extents everywhere in life. They are aluminum (Al), arsenic (As), lead (Pb), manganese (Mn), and mercury (Hg). Selenium (Se) should have been added to their list because it has only a narrow beneficial range of safe dose-levels above which it is also a known neurotoxicant. Thallium, also not listed, is an additional potent neurotoxin but not commonly encountered by the general populace and, if so, is soon apparent [35]. This published list also included cadmium (Cd), not generally labeled in this manner. One publication has reported a correlation for child neurobehavioral development in five-year old children in rural Bangladesh with Cd levels [36]. Whether this is valid awaits more detailed studies when more extensive testing might exclude other possible neurotoxicants that might be biasing the data. Generally, cadmium is regarded solely as a developmental modifier influencing birth statistics, such as baby weight, length, and head circumference [37]. It can be found in brain autopsies, but is not considered a neurotoxin at present [38,39]. As a result, the main elemental inorganic neurotoxicants of concern to the general public center on Al, As, Hg, Pb, Mn, and Se chemistries and merit in depth examination. The first four of these are non-essential to the body and serve no bodily purpose. Animals do appear to have some need for As, but a human requirement still remains debated and uncertain. Mn and Se are known to be essential micronutrients. Mn has a high level for toxicity in humans and cases of death are rare [40] and, as mentioned, Se has one of the narrowest ranges for human accepted needs, displaying dietary deficiency (<40 µg/day) but toxic levels (>400 µg/day) [41]. Reported deaths mainly center on supplements administered to animals, particularly horses, in areas where Se is at low levels in soil and grains [42], and are rare for humans. 

The published reference list mentioned above contains many more organic and organo-metallic compounds that also are known neurotoxicants. These are certainly hazardous to humans and cannot be readily disregarded. However, they do differ from the inorganics in generally being man-made and are less commonly encountered in normal human diets, but are present in many dermal or inhalation commercial products. They are also found in agriculture or food processing usage, but to be connected with global epidemics there are further criteria. For example, usage has to be global and newly-implemented in recent decades. Of course new pharmaceutical drugs are appearing annually. However, pre-release testing and initial prescription usage hopefully should indicate problems locally. Additionally, they generally have well-defined specific applications or purposes. As a result, problems generally should be reflected on local or small geographic scales and not be dispersed globally. This was not the case for DDT, of course, which was finally identified as carcinogenic and fortunately not a neurotoxicant for humans. Concern, generally with organic forms, relates more to their being simple toxins or carcinogens, and the public is at risk in this regard. However, it is almost like the drinking of caffeine in coffee: even though this is listed as having neural effects on animals, the risk is accepted, even though it might still have long-term problems for humans. Consequently, if any effect is not sufficiently pronounced some risk is accepted by the public. However, as a result of innumerable studies [43,44], any general global neurological danger to the public from known neurologically-labeled organics is not evidenced at present in the extensive medical literature in connection with these neurological illnesses considered herein. However, birth effects can be noted in other aspects, such as birth weight or head size. Those compounds that have become well-labeled as possibly producing bodily effects are more tightly regulated and are listed and known by the practicing medical profession [45]. For all that, whether some of the unlisted organic chemicals can aggravate or have a minor secondary contribution to the ever human body-burden certainly remains of concern and it would not be at all surprising if some do. One recent review did suggest certain organic pesticides might trigger ADHD in children [46]. Another Japanese study of 322 pregnant women noted 87 biphenyl and dioxin-type organic toxins present in their blood [47]. Although at low levels, such organic chemicals are becoming ubiquitous in humans and whether they induce low-level effects is difficult to measure. This becomes the dilemma. In fact, at what level does an organic toxicant, or mixture of them, become dangerous? This remains largely unchartered waters. 

The field of neurotoxicology, when relating to humans, remains an inexact science, especially for these chosen inorganic elements in this study, and is fraught with difficulties. Since human testing remains minimal and difficult for obvious ethical reasons, the bulk of the data have to be obtained from animal studies. In addition, significant other complexities abound. In humans, most toxicants come mainly from diet, with dermic or inhalation routes more a concern in the industrial workplace environment. Additionally, the toxicants can include a wide range of molecular forms that are generally absorbed to varying degrees. Such complexities introduce approximations particularly in scaling from the animal data to humans. This then converts to a toxicity unit of the form toxicant mass/kg human body weight/day. It then has to be scaled further to produce equivalent biomarker levels for medical testing of a preferred body sample, for example, generally for blood, hair, or urine concentrations. As is acknowledged, these conversions are not exact and a compensation factor for the expected errors in these approximations is invoked for an acceptable minimum risk level (MRL) to be recommended. For these elements a lowering generally by two orders of magnitude is preferable, if possible, below the laboratory-implied onset of toxicity, either the NOAEL (no observed adverse effects level) or the slightly higher LOAEL (lowest observed adverse effects level). In other words, to the best of our abilities, when also coupled to any auxiliary human data that may be available, the location of these actual toxicity onsets become reasonably established, but remain with possible errors depending on their source data. They form the basis for the then-recommended lower MRL biomonitoring values that are suggested with a built-in safety buffer of a possibly 10- to 100-fold margin, if this appears reasonable and acceptable. With the neurotoxins, this is the best that can be done at present, but MRL values are not rigid or precise due to the approximations or considerations made and can be modified over time with additional observations. This remains somewhat more unsatisfactory, especially for young children and for pregnancy, where the toxicity level itself may become more uncertain, as well as the scaling models. Questions concerning fetal risk from a growth of being less than a gram in the first eight weeks, to final birth weight are not satisfactorily addressed through a lack of knowledge. Prudence for life should, of course, always have body neurotoxicant levels as low as possible and especially throughout pregnancy. As will be noted, the fetal risk has to be high in all pregnancies and a choice of having low neurotoxic levels may generally not be available. 

For any one neurotoxic element, the level of toxicity generally varies from compound to compound, whether inorganic or organometallic, whether water soluble, or with a differing degree of absorption by the body [48]. Some factors also arise concerning valency, particularly for arsenic and mercury. Nevertheless, due to the more involved aspects of monitoring this valence distribution (speciation), particularly in drinking water for arsenic content, it is generally common to simply measure and quote the total amount. However, in fish, arsenic is largely present in a non-toxic organic form (arsenobetaine) that the body cannot metabolize [49]. Mercury is highly neurotoxic in organic forms, such as the methylmercury in fish [50], and thimerosal still used in some vaccines [51,52,53]. However, elemental Hg from tooth amalgams, for example, and most of its divalent inorganic forms normally encountered, are more toxic to the body than the brain. This is due to their difficulty in passing through the blood-brain barrier. Additionally, insoluble cinnabar, HgS, and monovalent calomel, Hg_2_Cl_2_ are essentially non-toxic. Nevertheless, by measuring the total As or Hg levels, normally done by several biomarkers, either involving blood, serum, urine, or hair sampling, although not precise and probably in error with regards to overall neurotoxicity, the results do err in the direction of overestimating the suggested body toxicity. A follow up, then, is an option to better analyze the situation. 

### The Neurotoxic Six Metal Elements and Their Compounds 

Although it is the responsibility of health agencies, such as the World Health Organization in Geneva, to establish these MRL (minimum risk level) values, they still remain uncertain and, in some cases, not exactly established or known. Aluminum is one important case for which values that exist still relate to specifically oral ingestion. Examined in great depth in recent decades, this centered mainly on evidence of toxicity from the now-replaced use of aluminum phosphate binders in previous dialysis treatments of kidney failure cases. Studies, then, for such oral ingestion, implied an MRL of 1 mg Al/kg body weight/day [54]. A significantly high level, indicating little risk. However, consumed in such a manner, intestinal absorption is known to be extremely low in the 0.1–0.6% range. Consequently, the recent introduction of aluminum hydroxide as the dominant adjuvant in many US vaccines has now modified the situation and requires renewed studies. Consequences of such inoculations have been analyzed far less, but one very extensive review now accepts that the levels of absorption by the body will be much higher [55]. Additional studies have suggested that this alone is a high medical risk for neurological complications [56,57]. In addition although the documentation concerning the known ingress/egress transport across the blood-brain barrier (BBB) remains hazy for Al, it has been shown to occur [58]. Isotopically-labeled ^26^Al studies effectively established this with rats suggesting long brain half-lives with indications of its presence also in fetal rat brains [59]. Additionally, intravenous feeding of preterm infants with commercial food products has long been of concern, as well as breast milk, for the possible contaminating Al content [60,61,62]. With any MRL, the unit contains “per kg body weight”. As a result, when applied to children this obviously increases in importance. Of the neurotoxicant inorganic elements, the risks associated with aluminum remain the least certain, its chemistry being predominantly inorganic, and its possible connection with old-age related consequences still remain very heavily debated [63]. Interestingly, aluminum also has recently been blamed as a risk factor in male infertility [64]. 

Although long known to be a serious neurotoxin, it is only in recent decades that lead’s anthropogenic presence has been more seriously considered. That leaded gasoline, lead in paint, in copper pipe solder, and crystal glass were extensively promoted is now considered unbelievable by many environmentalists. This is especially so with the World Health Organization (WHO) reporting that 143,000 children died globally in 2004 from lead poisoning. No meaningful MRL values are quoted for lead, but extensive toxicological reviews are available [65,66]. The accepted consensus is that any level of lead is unhealthy [67]. However, because a zero-value is not practical with lead’s ever-presence to some degree in the natural environment, it remains that less will always be beneficial and remain an objective purpose. One study of children in 1972 in New Zealand monitored their blood level content. It now reports, 45 years later, that early exposure does have long-term ramifications [68]. Additionally, regrettably, in cities such as Karachi, Pakistan, pregnant women, newborns, and children are still commonly experiencing blood values today in the range 100–500 µg/L when values <50 µg/L are desirable [69]. Due to lead’s continued presence in many locations, only temporary MRL values currently tend to be in use, mainly as a coarse measuring indicator for doctors, and are gradually being lowered as it becomes possible with environmental improvements. Ultimately, as there is a return to low natural levels in the normal environment, a firm and more meaningful MRL target may become possible for lead and imply a safe level for minimal neural damage. 

Mercury, another potent neurotoxicant documented through the centuries has also come to the forefront in recent decades. This is mainly through concerns due to its presence as the organometallic thimerosal in vaccines, and also now especially through the added recognized risk associated with the presence of methylmercury in most fish varieties. Environmental pressures have been severe to the extent that mercury is no longer mined globally or used commercially. All its applications in most countries have been banned and remaining anthropogenic sources tend to be solely the unregulated emissions from coal combustion and some cement production, medical uses, its diminishing application in dentistry, and some continued use in artisanal gold mining. Significant efforts by the United Nations and others now are attempting to control such use in mining [70]. Its toxicology is well documented [71,72]. Additionally, monkey blood and brain studies have clearly confirmed organic mercury’s ability to enter the brain. For ethylmercury, from thimerosal, about one-third becomes inorganic and two-thirds remains organic. Therein, the brain half-life for the organic fraction is about 14 days, but 24 days for the total loss of mercury. For methylmercury, it mainly remains as such with a residual brain half-life of about 60 days [73,74]. This had been shown earlier with pregnant hamsters for methylmercury using isotopically-labeled ^203^Hg added to their diet, finding it in both the brains of mother and fetus [75]. Placing more emphasis on studies considering people on high fish diets, coupled to extensive animal studies, an NOAEL Hg blood level of 60 µg/L was suggested. This led to the U.S. Environmental Protection Agency quoting a recommended MRL blood value of 5.8 µg/L [71], only 10-fold lower, which is now in common usage and will be discussed further below when data are presented.

Extensive toxicological reports have been published for arsenic and suggest an MRL of 5 µg/kg body weight/day [76], also only 10-fold below its LOAEL value. In California under a State Proposition 65, listing controlled chemicals, this limit has been reduced now to 10 µg/person per day, but the new emphasis relates more to the risk of cancer rather than neurological damage. In areas of the world where water can contain concentrations of As > 50 µg/L it is obvious that small children will be at significant risk for both cancer and neural damage. As indicated already, arsenic’s ingestion rate can be complex with biological (organic) forms in seafood generally being non-toxic [49]. Its ingestion is predominantly oral, resulting mainly from drinking water sources. In many parts of the world this is a dominant concern. Average U.S. dietary needs, if any, remain unknown but have been suggested as 12–25 µg/day approximated from animal studies [77]. The LOAEL toxic level is considered to be quite well established in this case. As a result, although arsenic may be of less concern in more developed countries, arsenic in drinking water and edible crops requires close management elsewhere [78,79]. Arsenic is cleared quickly from the blood and generally hair or urine samples are a more meaningful biomarkers of prolonged exposure. 

Manganese differs from the above four in being an essential micronutrient to the human diet. At higher dose rates, though, it does become neurotoxic and is known to influx the brain [80,81]. It has been studied extensively in animals and humans, but a firm MRL value remains uncertain. A major toxicological review quotes an interim guidance value of 0.16 mg/kg body weight/day based on a 70 kg adult and an average of about 11 mg/day ingestion [40]. Its use in fungicides, and now as a fuel additive, have raised few concerns thus far. It is present in many foods and can be significant in teas and some herbal drinks. However, potential risks to children and a fetus in pregnancy are labeled as important, but appear to remain a low priority by agencies in spite of possible reported drinking water effects and other concerns [82,83,84,85]. No MRL value is quoted for pregnancy. 

Selenium, although a potential neurotoxin, is now being recognized as an extremely important element in the body. The fact that DNA is programed to produce about 26 different seleno-proteins in the body lends support for such a consensus. These now appear to be major chelating antioxidants for cleansing the body and particularly the brain of toxicants. They are capable of removing the above five mentioned neurotoxins, namely Al [86,87], As [88], Pb [89], Mn [90], and Hg [91,92]. Selenium has a significant biological presence in the body. Dietary intake has now been raised from earlier suggestions to a range of 200–300 µg/day for adult good health [93,94], but which is not too far below the toxic level of >400 µg/day [41,95]. Deficiencies certainly need to be satisfied, but abundant levels should not be supplemented. 

## 3. Blood Level Neurotoxins in the General Populace 

The advent of improved monitoring capabilities has changed science and medicine, particularly those based on the inductively coupled plasma method in either its optical spectral emission or its more sensitive mass spectrometric modes. It has introduced a flurry of large scale testing programs around the world aimed at obtaining databases and establishing average baseline values of innumerable compounds in blood, hair, and urine or body tissues. This has helped in better-recommended minimum risk levels, particularly for toxins and neurotoxins, and has enabled legislated steps concerning safety levels in the workplace. The first surprise found in all these human media results was the richness of trace chemicals present in the body. One study examined 27 metal elements in blood and serum samples, finding all of these easily, except four, within the level of detection [96]. More ambitious programs have included anthropogenic organic compounds, such as polychlorinated biphenyls (PCBs) and other persistent organic pollutants, and have enlarged this list to display the presence of over a hundred [44,97]. In worldwide blood samples, all the main neurotoxin and toxic inorganic elements are invariably observed. However, analytical sensitivities now are such that the question is not whether something is present but, more importantly, its magnitude. This is where the value of an average or minimum risk level arises and can better guide a medical examination. However, from a scientific point of view, there is one interesting aspect in these large surveys that has not been as strongly emphasized. This concerns not solely the average and its relationship to appropriate MRL values, but more so the spread of the distributions for any of these elements or compounds. It is clearly apparent that people differ significantly, even for groups that have very similar diets and lifestyles. Distributions quite generally can be broad and it is obvious that such variations reflect that some people can shed a toxin more efficiently, while others tend to be retentive. 

To examine this aspect of broad distributions further, numerous large blood testing databases from around the world that included neurotoxicant values have been re-examined. Concerning the general populace in various countries, the data for some are compiled in Table 1. Listed are approximate distribution averages, together with maximum values, some estimated from the 95–98% percentile-level of the distribution, and some measured. The databases selected have been chosen from the very many such tabulations only to display these possible variations and trends. Their exactness and reliability are of minor concern here, but they are chosen so as not to be perturbed by any unique environmental aspect, such as a heavy industrialization bias. In all, it was noted that male/female levels for these elements were very similar, except for lead, for which male levels are generally slightly larger. The first thing observed is the very limited data available for blood aluminum levels. This results from the previous lack of concern with dietary or orally-consumed aluminum, which is minimally absorbed by the body. However, now that aluminum hydroxide has become a major adjuvant in most US vaccines, it should certainly be added to future surveys as increased concerns about its use have been raised [55,56,59,98,99]. Those few data that are available do not discuss this potential bias and cannot be realistically analyzed. They do clearly indicate its presence. 

As mentioned above, the establishment of satisfactory MRL values are important mainly as a rough guide, but do remain uncertain in several cases. Those for arsenic, manganese, mercury, and selenium have a basis, as described already, from animal and some human data. They are in use at major medical blood-testing centers. The value for mercury listed in Table 1 has been the main recommended US value now for some time, but other countries have modified it, for example, in Germany to 0.8 [115] and two US testing centers use values of either 9.0 or 2.0 µg/L. These together cover an 11-fold range and further illustrates the varying uncertainties and safety risk assessments. As indicated, the case for Al remains very uncertain, with the US Mayo Clinic suggesting the value in Table 1, but another facility stating <30 µg/L [116,117]. 

The situation remains difficult with Pb, with MRL values being suggested targets rather than being based on the realistically-observed measurements in adults. Germany again suggests a lower value of 35 µg/L for children. However, a major guide at present is to try and maintain the populace below 50–100 µg/L as much as possible. Generally, trends are drifting lower as communities continue the endeavor to minimize this particular hazard. The difficult question is how much larger does an observed value have to be above a suggested MRL level before chelation becomes recommended. At present, this is often when toxic effects are visually present. In cases such as the 2000–2004 crisis of lead in the drinking water of Washington, DC, the consequences are still being documented [118]. Small children suffered from poisoning and there was a high incidence of miscarriages and fetal deaths even for blood levels of 50 µg/L, a value still evident in numerous parts of the world.

**Figure 1:**
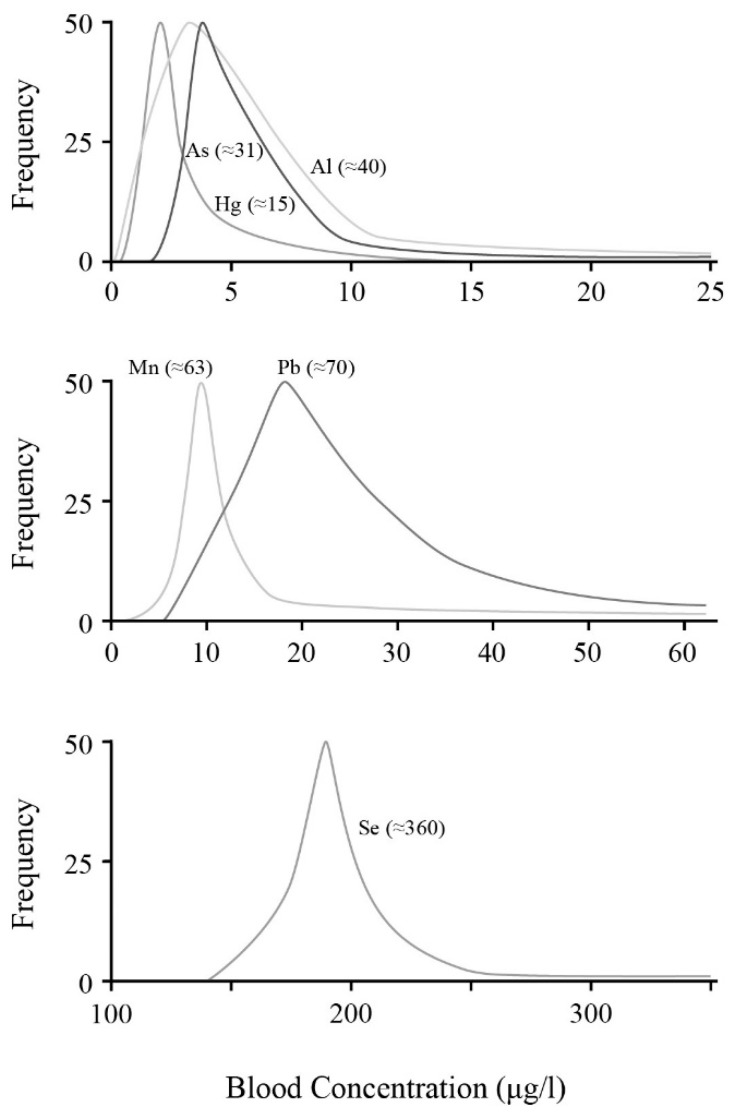
Samples of blood concentration/percentage-distribution surveys for adult populations for the six neurotoxins. Approximate maximum values are given in parenthesis indicating the upper value extremes and tail lengths reported for outlier cases. Displayed values are taken from As [106]; Al [103]; Hg [107]; Mn [112]; Pb [103]; and Se [114].

Several of these distributions are plotted in Figure 1 to better illustrate the distributions. Generally these are statistically tabulated, but not usually visually drawn in this manner. To some degree this is because of the commonality, and the major interest in average values and their standard deviations. For example, in the present case, a comparison of the lead and mercury profiles shown here do mirror those portrayed previously in a totally differing recent Polish survey [119]. 

Although basic shapes may stay similar from one survey to another, average values will vary with geographic locations. However, a common aspect is the long outlier tails that can extend well beyond the 95 percentile level and, in most, cases many-fold above the average value. The profile for selenium appears to differ considerably from the others in seemingly being tightly controlled between its extremes in numerous surveys. Although its average can vary, the shape seems to be quite common. Nevertheless, studying all these surveys, values can stretch quite significantly above the average, and sometimes do extend to ten-fold, or more, above the MRL value. In other words, the toxicological efforts to safeguard society appear reasonably valid for the general populace. However, in the outlier cases, these approach and can venture into the toxicity curve region. For example, in one US survey with 1800 blood-lead samples, 4.8% of the distribution was ≥50 µg/L, including 12 women of reproductive age; eight samples were ≥100 µg/L, and two were ≥250 µg/L [113]. Extreme examples are commonly seen to exist. 

In addition to considering the neurotoxins individually, this, of course, overlooks an obvious question: How does the human body simultaneously respond to multiple toxins? For the majority of people, observations do confirm that the body mechanisms appear to be sufficient to control most neurotoxins. However, as mentioned below, synergistic factors between various trace metals have now been reported. Additionally, the fact that society is presently documenting mercury poisoning cases in adults illustrates that the above safeguards are failing at times [120]. Additionally, there is no measure available to indicate only slight neurological effects that might be accumulating over time. 

## 4. Blood Level Neurotoxins in Pregnant Women 

**Figure 2:**
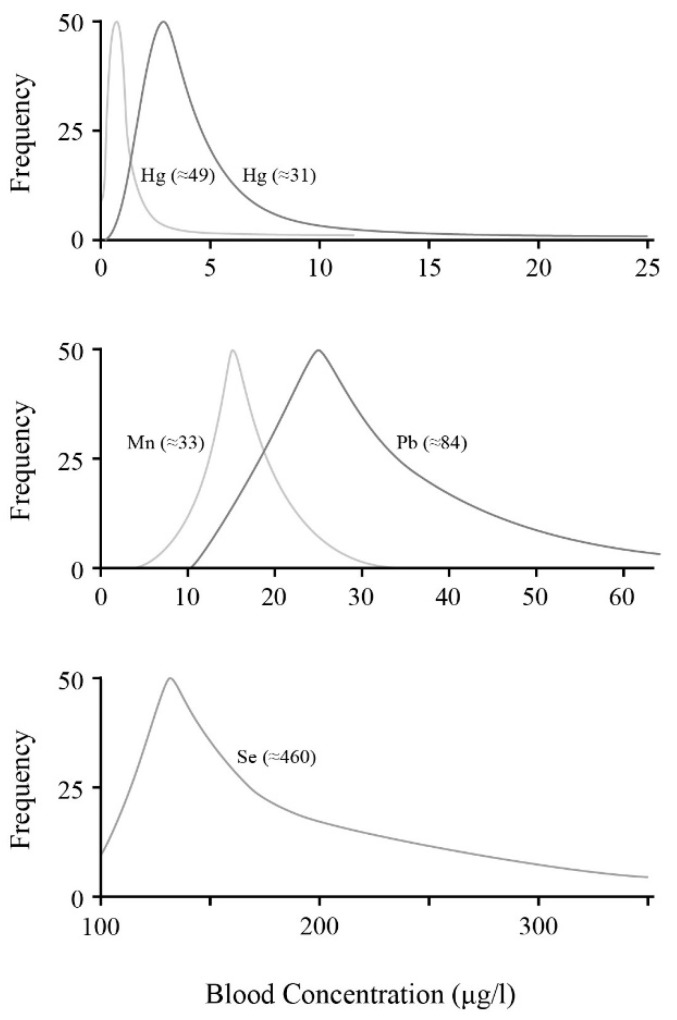
Samples of blood concentration/percentage-distribution surveys of pregnant women for four of the neurotoxin metals. Approximate maximum values are given in parenthesis indicating the upper value extremes observed. Displayed values are taken from Hg [134,135]; Mn [130]; Pb [138]; and Se [127].

Due to the known major risks concerning pregnancies, even more extensive databases have been generated measuring toxicants in a mother’s blood during the stages of pregnancy and in the baby’s umbilical cord at birth. A similar sampling of data are listed in Table 2. Again, this shows an average and a suggested maximum value for each statistical distribution. Some of these databases provide information on the full distribution and are further illustrated in Figure 2. 

As already noted in Figure 1, the maximum value recorded or estimated is also shown in parentheses. Comparing Figure 1 and Figure 2 is difficult in that quantitative magnitudes can vary significantly from country to country depending on their location and environment. Nevertheless, the distributions can have similar shapes. All display the steep rise at lower values where the majority of samples are located, and then a more gradual fall-off representing the fraction lying above the average and with wider variability. In Figure 2, two profiles are displayed for Hg to indicate how differences may occur from one survey to another. However, even in these cases, although one is narrower, it has the longer extended tail. Other than for Se, there really are no noteworthy differences between the general or pregnant women distributions.

Even in pregnancy, average values do not exhibit drastic reductions, even though one might expect greater diligence now as expectant mothers are becoming better warned of dietary risk factors. Whether the noticeable change in distribution for selenium with pregnancy seen in Figure 2 is meaningful requires confirmation, but the distribution seems to broaden and extend to much higher values. Whether this suggests some importance for selenium’s chelating, anti-oxidant role remains too speculative. The main aspect of the current analysis is to illustrate the extension of outlier values that can stretch many-fold above the average and is common for all these toxicants. Of course the blood concentrations of expectant mothers will be biased already at conception. Whether their levels can be reduced significantly by dietary changes requires testing. As a result, the major conclusion from Figure 1 and Figure 2 is simply that everyone, pregnant or not, can have noticeable levels of these blood neurotoxicants. Moreover, at present, there appear to be no major differences seen between the general populace and pregnancy. A recent extensive review summarizes the blood levels during pregnancy for Pb, Hg, and Cd in various surveys taken after 2000. The review stresses the need for much better MRL values, especially for pregnancy. However, it also reiterates that, realistically, there are no safe blood-level values for any of the heavy metals during pregnancy [137]. 

Placental protection of the fetal blood has been very extensively studied. A recent review has been published of the birth-cord blood to maternal-blood concentration ratio of innumerable elements and compounds from more than 100 studies [139]. For the six principal neurotoxicants discussed here, the placenta is seen to be very pervious. For lead and selenium this ratio is close to unity, arsenic is slightly below unity, but rather surprising, mercury and manganese are two- to three-fold larger in the fetal blood. That for aluminum remains unclear, but the cord blood was high in one study [122] and the ratio might have been about a half or slightly protective in others [140,141]. Nevertheless, isotopically-labeled studies on pregnant rats have clearly identified Al transfer into the brain of their fetus [59]. Clearly, the placenta is incapable of protecting a human fetus with regards to neurotoxicants or most other heavy metals. 

As noted above, toxicology utilizes in its unit, “quantity per body-weight”. The fetus starts from a minimal body-weight and is only about 1 g at eight weeks. As a result, and with this minimal placental protection, the important question is “what is the safe MRL for a fetus and how does it even survive?” Few medical papers appear to have addressed this aspect. One that did analyzed the situation primarily for mercury, concluding that the fetus was always at very high risk [142]. This would appear to remain valid and the only conclusion to be logically drawn is that the fetus must have a very efficient blood/brain barrier from initiation and/or additional protective mechanisms as yet unknown. 

## 5. Why Now?

The fact that society is now displaying changes in neurological health appears undeniable. Care centers are appearing everywhere and publications concerning neurological health are more extensive. The general consensus is that this is not a primary result of genetic changes or improved diagnosis, but more probably results from the environmental complexity of our modern age. Present-day monitoring capability has illustrated in the results listed herein that human bodies are now constantly being contaminated by dietary and commercial product ingredients. Although society is continually introducing stricter controls, and significant steps forward are being made in some areas, at the same time life-styles are constantly changing. However, if neurological epidemics are a consequence, this has to be reflected by some societal change that has occurred in recent decades. 

To affect pregnancy on a global scale this has to involve a change of ingestion of neurotoxicants either from the air, diet, or medically. In normal life, the three-dimensional dilution of pollutants in air can generally eliminate any noticeable levels of neurotoxicant intake directly from this. As a result, the logic suggests a necessary increase must have occurred in the diet or some medical protocol. This implies that some people are going beyond their body’s limits that are defined by their individual genetic nature. An additional factor, not yet addressed, is that our measured toxicology data mainly relates to the safety aspects of substances when considered singularly. More and more, in life and science, the addition of multiple factors coupled to aspects of synergism are becoming evident. This has been studied medically in the body mainly in how its complex balances may be modified by added changes. Some aspects of synergistic effects for mixtures of toxins have been noted in human body organs [143], but very few studies are yet available to examine such effects with neurotoxicants. One such important examination looked at long-term exposure of mice to low doses of Pb, Hg, As, and Cd individually and in their mixtures [144]. This extended and confirmed earlier work on rats with Pb, As, and Mn [145]. These very clearly indicated that the neurotoxin elements could be affected by other toxins or even other essential metal elements in a synergistic manner, certainly enhancing their presence in the brain of animals. This has now generated interest in the possible mechanisms of such interactions [146]. In other words, from the point of view of toxicology of the brain, there remains a depth of uncertainty. It is partly a complex chemical kinetic problem that depends on brain ingress and egress rates, lifetimes in the brain, and how quickly neutralization or sequestration occurs for anything that may be held there. Additionally, on rates of damage and whether this can be repaired [147], or replaced by surplus circuitry, very little such data yet exist, except some for particularly organic-mercury in animals. In that case, research has now introduced the aspect that elevated levels of Se do appear to protect the brain against Hg in mice and rats, and that neutralization mechanisms in the brain may, in fact, be quite rapid [92,148]. Moreover, one study on rats even reported a reversal of Hg toxicity when their diet was switched to one that was Se-rich [149]. Furthermore, some Japanese residents who consume significant levels of whale meat and display dangerously high levels of methylmercury in their hair and blood, show minimal neurological damage. On analysis, it is apparent that their blood Hg/Se atomic ratios are always less than unity and it is concluded that the Se may alleviate their risk [30]. Many other studies are now stressing this possible importance of the Se/Hg atomic ratio in fish [150,151]. Additionally, numerous new studies now are underway concerning the benefits of Se [152,153], during pregnancy [154], for the elderly [155,156,157], and a possible role for this in Alzheimer’s disease treatment [158,159,160,161,162,163]. 

It is evident we need knowledge of these six neurotoxicants in the body, and lifestyle control is necessary to maintain them at a safe level, not only individually, but probably their summation. Since the epidemic of neurological illnesses has mainly occurred in the last 30 years, the causal effect has to be compatible with such a time-frame. One such exercise was recently completed, relevant particularly to the US [164]. It indicated that the three- to four-fold increase in vaccination schedules, coupled to the introduction of social “Japanese-style” sushi additions to the diet in this same time period could be excessive. When considered together they closely correlated with the growth of autism. The present analysis lends supports for such a conclusion. Additions of other minor sources with possible synergistic contributions can readily overburden the body’s ability to remove such toxicants in some fraction of the human population. In pregnancy, the fetus is even more vulnerable due to its small size. Additional concerns are now suggested by the increasing rates of miscarriages, not only in industrialized areas, but also in sub-Saharan countries, all being linked to the presence of heavy metals [118,165,166,167,168]. Another aspect is the possible transport in semen [169], and the general role of trace metals in the fertility of both males and females [170,171]. Additionally, the risk of small accumulation rates in the brain over a lifetime can no longer be ignored. 

## 6. Conclusions

Societies often are content with their status quo until a problem arises; then it has generally been normal to look for the cause, usually singular, and correct it. With the current epidemic of neurological illnesses, both in the young and the old, this has been increasing rapidly over the last 20–25 years with no resolution. A tremendous wealth of medical research has questioned the potential roles of known neurotoxicants, organic or inorganic, indicating that not one of these clearly stands out. Vaccines have been extensively questioned [51,52,53,56,172], but testing has always appeared to confirm their safety. However, it is true that some disturbing evidence is apparent otherwise. One report noted the enhanced rate of miscarriages in the US during the 2009/2010 influenza vaccine period [173]. For the first and last time, pregnant women were given two different influenza vaccines instead of the normal one during any trimester. They both contained thimerosal. The analysis showed that miscarriages that year increased by more than an order of magnitude compared to earlier or later years. It remained unexplained. The attempted emphasis on a single cause has now changed, and numerous studies other than this one are beginning to suggest the possibility that combined factors do enhance the risk involved, with possibly additional synergistic effects [146,164,174]. The present study, with a more chemical, scientific approach, extends this further, and now appears to clearly exhibit that this has the potential, in some cases, to overload both humans and fetuses with neurotoxicants. It is very plausible and satisfies the necessary criteria. Consequently, if the risk of neurological damage is to be reduced, changes will have to be made to various lifestyles. In fact, even three years ago, the United States EPA indicated grave concern over the increasing chronic methylmercury exposure from fish consumption [175]. Moreover, several groups have already realized the significant value in taking such a step by analyzing cost and intellectual benefits that would result from reducing neurological damage [176,177]. Nevertheless, this will be difficult. For example, obstetricians today are faced with assessing the risk concerning a fish diet during pregnancy [178,179]. This has become an even more difficult risk analysis calculation especially if the moderating aspects of selenium are correct. Consequently, although the average mercury content of most fish varieties have now been listed, they may need repeating, to list the corresponding Se content [180]. Additionally, governmental changes and directives are clearly needed concerning vaccines and any neurotoxic content. Canada is one country that already has taken action, particularly with regard to safer vaccines for pregnancy [176]. 

To be prudent, one important step would be to initiate testing to establish the baseline values of these neurotoxicant metals in all women of child-bearing ages. In some cases waiting until pregnancy may be too late and this might also reduce the high rates of miscarriages that now are reported. A one-time testing when young could be sufficient for most women and clearly identify those with levels controlled by diet or a genetic susceptibility. This would significantly reduce anxiety concerns in all women and be of value to health. In cases where higher level susceptibilities were found, adequate modification to diet could be implemented [181]. Men, would also probably like to know their category of genetics, whether they were below or above average with regards to retention, and this could possibly aid in resolving fertility questions. It would remove a presently unknown aspect of our body that exists. If necessary, sources of drinking water might be analyzed for content and treated. In the US, lead might be of concern in older homes due to old paint, the solder used on copper pipes, or even lead piping in some communities. Additionally, the medical profession has to reassess its current general vaccination program. Although there is no denying this has been a tremendous success, it has now grown three- to four-fold in size in the last 30 years and has become excessive. It is twice as large as any other country [182]. By six years old, a child in the US can have 35 inoculations that increase to a total, ten years later, of about 45. It can no longer be denied that this is contributing to body-burden, especially if administered in multiple doses at the same time. Such a practice has to be considered dangerous, irresponsible, and certainly should be ended. Furthermore, development and use of alternate adjuvants for neurotoxicant-free vaccines is critically needed. 

Additionally, a very difficult aspect that is still increasing is the extent of certain fish in diets. Its nutritional value is beyond question, but the risk, particularly for young women, is severe, requiring guidance. Moreover, this risk is present for all children, and even for adults. Even with the brain’s protective mechanisms, and possibly adequate Se content, the extent of minimal damage from even small doses of neurotoxicants, such as methylmercury, remains unknown. Many toxicologists are now suggesting the true safe-level for any neurotoxin is as close to zero as possible. The assumption that the body is resilient and can accept a certain level of abuse is, at best, unwise. Potential biomarkers are now available to better minimize risk before the onset of damage occurs and should be considered very seriously, especially as life-span increases [183]. Low-level goals are certainly prudent. The toxins and neurotoxicants, with several exceptions, have always been in the human diet from natural sources, however, we have no measure in history to indicate what spectrum of damage they may have created. It may still be hoped that the body can normally control low levels without long-term ramifications.

## Figures and Tables

**Table 1:** Typical average values and observed upper limits of the six neurotoxins monitored in blood samples in various adult population surveys, µg/L.

	MRL	Average (Maximum Value), Country				
Al *	<6	37 [100] Turkey	4.6 (≤17) [101] USA	7.1 [102] Korea	4.3 (≤11.8) [103] France	
As	<12	1.9 (≤7.1) [96] France	5.9 (≤41) [104] Norway	7.0 (≤56) [105] Bangladesh	4.1 (≤31) [106] Brazil	0.8 (≤18) [107] Finland
Pb	50–70	2.7 (≤131) [108] Brazil	25 (≤65) [104] Norway	2.4 (≤245) [109] Ghana	23 (≤54) [103] France	17 (≤146) [107] Finland
Mn	<18	18 (≤88) [110] Mexico	8.9 (<17) [111] Italy	9.9 (≤62) [112] USA	13 (≤119) [106] Brazil	1.5 (≤42) [109] Ghana
Hg	<5.8	2.7 (≤36) [113] USA	1.7 (≤5.1) [96] France	4.0 (≤13) [104] Norway	1.4 (≤12) [108] Brazil	2.5 (≤15) [107] Finland
Se	70–130	110 (≤142) [96] France	95 (≤153) [104] Norway	123 (≤222) [105] Bangladesh	190 (≤253) [114] USA	104 (≤245) [107] Finland

MRL: Suggested minimum risk levels; specific references are in squared parentheses. * No reliable data are yet available containing vaccine effects.

**Table 2:** Typical average values and observed upper limits of the six neurotoxins monitored in blood samples from various pregnant women surveys, µg/L.

	MRL	Average (Maximum Value), Country				
Al *	<6	65 (≤860) [121] Australia	11 (≤28) [122] Jamaica			
As	<12	0.4 (≤8.6) [123] USA	1.9 (≤16) [121] Australia	2.1 (≤37) [124] Croatia	1.2 (≤33) [124] Italy	0.6 (≤5.8) [125] Belgium
Pb	<50	8.9 (≤77) [123] USA	29 (≤260) [126] Saudi Arabia	45 (≤137) [127] China	77 (≤287) [128] Mexico	10 (≤22) [129] South Korea
Mn	<18	9.1 (≤50) [121] Australia	15 (≤33) [130] USA	12 (≤40) [125] Belgium	66 (≤304) [131] China	13 (≤34) [132] Canada
Hg	<3.5	8.0 (≤16) [133] Poland	2.4 (≤40) [124] Italy	3.7 (≤31) [134] Mexico	0.9 (≤49) [135] Saudi Arabia	11 (≤241) [136] North Canada
Se	70–130	102 (≤374) [121] Australia	117 (≤229) [124] Italy	90 (≤182) [124] Croatia	157 (≤456) [127] China	271 (≤357) [136] North Canada

MRL: Minimum risk level (not clearly defined at all for pregnancy and the fetus). Specific references are in squared parentheses. * No reliable data are yet available containing vaccine effects.
